# Supplementary material for: A proposal for leaky integrate-and-fire neurons by domain walls in antiferromagnetic insulators
Source: Sci Rep. 2023 Aug 17;13:13404. doi: 10.1038/s41598-023-40575-x (PMC10435549; doi:10.1038/s41598-023-40575-x)
Supplement: Supplementary file 1 — Supplementary Information 1. [file 41598_2023_40575_MOESM1_ESM.pdf]

Supplementary Information for "A proposal for leaky integrate-and-fire neurons by domain walls in antiferromagnetic insulators"

Johannes W. Austefjord,[1] Verena Brehm,[1,*] Serban Lepadatu,[2] and Alireza Qaiumzadeh[1]

[1] *Center for Quantum Spintronics, Department of Physics, Norwegian University of Science and Technology, 7491 Trondheim, Norway*

[2] *Jeremiah Horrocks Institute for Mathematics, Physics and Astronomy, University of Central Lancashire, Preston, PR1 2HE, United Kingdom*

In this Supplementary Information, we first demonstrate that the functionality of the proposed neuron is scalable. To prove that we use parameters of hematite, a prototype of two-sublattice AFM insulators. Additionally, we show that in our proposed set up, the imaginary part of the spin mixing conductance is not relevant.

I. EASY-PLANE HEMATITE

We consider AFM hematite (α-Fe_3O_2) above the Morin transition temperature where the system is in magnetic easy-plane phase. In Fig. S1, we present magnon induced domain wall (DW) motion for the easy-plane phase of hematite above the Morin transition, which is a prototype of orthorombic AFMs.

The motion is controlled by a magnetic field (position and duration indicated by orange area, to scale) with two opposite helicities (indicated by arrow). Two values of the bulk Dzyaloshinskii–Moriya interaction (DMI) D are compared (blue vs green line). This is analogous to the magnetic field controlled motion presented in the main text with the four-stage protocol. Note that the system is larger compared to the toy model presented in the main article, due to the DW width. The DW equilibrium position is at 2 µm.

As expected from our proposal based on our toy model parameters in the main text, both magnetic field helicity and direction (sign) of the DMI switch the direction of DW displacement. We choose an anisotropy profile $K(\vec{x}) = 10K_0\left[\frac{1}{L_x}\left(x-\mathcal{X}_0\right)^2+1\right]$. Note that the slope of the profile can be tuned even larger as it was discussed in previous studies [1]. The simulation parameters for hematite [2], are presented in Table S1.

In summary, our proposed neuron can be realized in AFM systems with generic orthorhombic symmetry. Excitation timescales should be tuned for each chosen material.

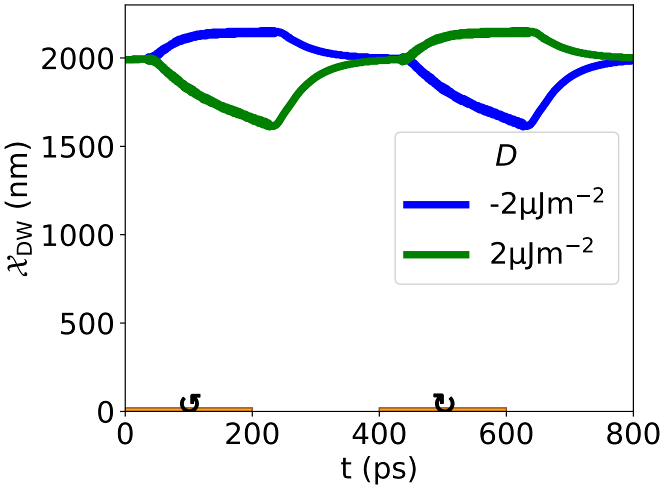

FIG. S1. Magnetic field controlled DW motion in easy-plane hematite.

* verena.j.brehm@ntnu.no

TABLE S1. Simulation parameters for hematite [2].

Quantity	Symbol	Value	Unit
Length of AFMI layer	L_x	3.0	µm
Width of AFMI layer	L_y	20	nm
Thickness of AFMI layer	L_z	4	nm
Grid size	a	4	nm
Exchange stiffness	A_{AFM}	76	$\mathrm{fJ\,m^{-1}}$
Homogeneous exchange constant	A_h	−460	$\mathrm{kJ\,m^{-3}}$
Easy-axis anisotropy constant	K_{easy}	−21	$\mathrm{mJ\,m^{-3}}$
Hard-axis anisotropy constant	K_{hard}	21	$\mathrm{J\,m^{-3}}$
Saturation magnetization	M_s	2.1	$\mathrm{kA\,m^{-1}}$
Gilbert damping	α	0.0003	1
Homogenous DMI coefficient	D_h	4.6	$\mathrm{kJ\,m^{-3}}$
Time step	Δt	2	fs

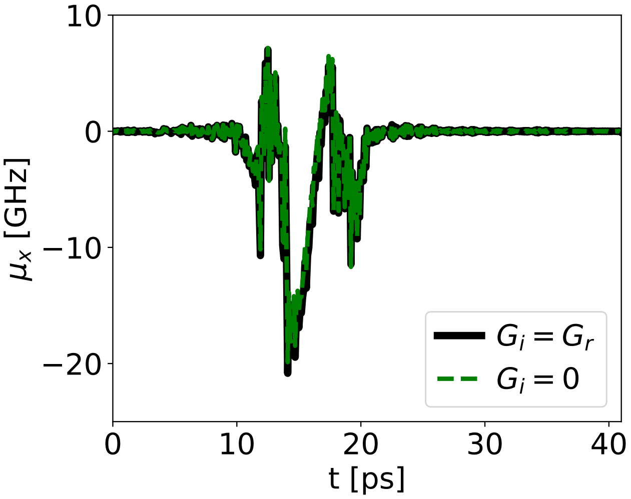

FIG. S2. Comparison of read out spin pumping signal including and not including the imaginary spin mixing conductance.

II. CONTRIBUTION OF THE IMAGINARY PART OF THE SPIN MIXING CONDUCTANCE

In general, the imaginary part of the spin mixing conductance is dependent on the quality of the interface between the heavy metal layer and the magnetic layer. This term is negligible for dirty interfaces. The spin pumping has the following general form [3, 4]

$$\boldsymbol{\mu}(t) := G_r^{\uparrow\downarrow}\big(\boldsymbol{n}(t,\boldsymbol{r}) \times \dot{\boldsymbol{n}}(t,\boldsymbol{r}) + \boldsymbol{m}(t,\boldsymbol{r}) \times \dot{\boldsymbol{m}}(t,\boldsymbol{r})\big) - G_i^{\uparrow\downarrow}\dot{\boldsymbol{m}}(t,\boldsymbol{r}), \tag{1}$$

with the Néel vector $\boldsymbol{n} = \frac{\boldsymbol{m}_A - \boldsymbol{m}_B}{2}$ and magnetization $\boldsymbol{m} = \frac{\boldsymbol{m}_A + \boldsymbol{m}_B}{2}$, where $G_r^{\uparrow\downarrow}$ and $G_i^{\uparrow\downarrow}$ are the real part and the imaginary part of the spin mixing conductance, respectively.

In order to check the qualitative and quantitative effects of including $G_i^{\uparrow\downarrow}$, we compare two extreme cases, i.e., $G_i^{\uparrow\downarrow} = G_r^{\uparrow\downarrow}$ (large imaginary part) and $G_i^{\uparrow\downarrow} = 0$ (zero imaginary part). As shown in Fig. S2, both read outs are the same, suggesting that the imaginary part of the spin mixing conductance can be neglected in our set up geometry.

REFERENCES

[1] L. Shen, J. Xia, G. Zhao, X. Zhang, M. Ezawa, O. A. Tretiakov, X. Liu, and Y. Zhou, Dynamics of the antiferromagnetic skyrmion induced by a magnetic anisotropy gradient, Phys. Rev. B **98**, 134448 (2018).

[2] O. R. Sulymenko, O. V. Prokopenko, V. S. Tiberkevich, A. N. Slavin, B. A. Ivanov, and R. S. Khymyn, Terahertz-frequency spin hall auto-oscillator based on a canted antiferromagnet, Phys. Rev. Appl. **8**, 064007 (2017).
[3] Y. Tserkovnyak, A. Brataas, and G. E. W. Bauer, Enhanced gilbert damping in thin ferromagnetic films, Phys. Rev. Lett. **88**, 117601 (2002).
[4] R. Cheng, J. Xiao, Q. Niu, and A. Brataas, Spin pumping and spin-transfer torques in antiferromagnets, Phys. Rev. Lett. **113**, 057601 (2014).
