# Supplementary material for: A proposal for leaky integrate-and-fire neurons by domain walls in antiferromagnetic insulators
Source: Sci Rep. 2023 Aug 17;13:13404. doi: 10.1038/s41598-023-40575-x (PMC10435549; doi:10.1038/s41598-023-40575-x)
Supplement: Supplementary file 2 — Supplementary Information 2. [file 41598_2023_40575_MOESM2_ESM.pdf]

Supplementary Information 2 for "A proposal for leaky integrate-and-fire neurons by domain walls in antiferromagnetic insulators"

Johannes W. Austefjord,[1] Verena Brehm,[1,*] Serban Lepadatu,[2] and Alireza Qaiumzadeh[1]

[1] *Center for Quantum Spintronics, Department of Physics, Norwegian University of Science and Technology, 7491 Trondheim, Norway*

[2] *Jeremiah Horrocks Institute for Mathematics, Physics and Astronomy, University of Central Lancashire, Preston, PR1 2HE, United Kingdom*

I. SIMULATION CODE

All data was generated using the BORIS software [1]. The simulation was set up and analyzed using the python file given below. A detailed description of the commands can be found in the BORIS manual. Simulation parameters in the python script (material parameters, excitation strength and duration, the injector and detector positions and widths, and the anisotropy profile) are just examples and can be varied. Data presented in the article was generated using simulation parameters presented in the article.

```python
from NetSocks import NSClient, customize_plots
from utils import utils
import os
import pandas as pd
import numpy as np
import matplotlib as mpl
import matplotlib.pyplot as plt
import matplotlib.pylab as pyla

font = {'size'   : 20}
mpl.rc('font', **font)


def Init(geometry):
    ns = NSClient(); ns.configure(True)
    print('init system in geometry '+geometry)

    ns.reset()
    Lx = 1000
    Ly = 20
    Lz = 4

    base_layer = np.array([0, 0, 0, Lx, Ly, Lz]) * 1e-9

    ns.setafmesh("base_layer", base_layer)
    ns.cellsize(np.array([4, 4, 2]) * 1e-9)

    ns.addmodule("base_layer", "anitens")
    ns.setparam("base_layer", "grel_AFM", (1, 1))
    ns.setparam("base_layer", "damping_AFM", (0.002, 0.002))
    ns.setparam("base_layer", "Ms_AFM", 2.1e3)
    ns.setparam("base_layer", "Nxy", (0, 0))
    ns.setparam("base_layer", "A_AFM", 1e-12)
    ns.setparam("base_layer", "Ah", -200e3)
    ns.setparam("base_layer", "Anh", (0.0, 0.0))
    ns.setparam("base_layer", "J1", 0)
    ns.setparam("base_layer", "J2", 0)
    ns.setparam("base_layer", "K1_AFM", (20e3, 20e3))
    ns.setparam("base_layer", "K2_AFM", 0)
```

* verena.j.brehm@ntnu.no

```
    ns.setparam("base_layer", "K3_AFM", 0)
    ns.setparam("base_layer", "cHa", 1)
    ns.setparamvar("K1_AFM", "equation",  "abs(x/Lx - 2/3)^2 + 1")

    if (geometry == 'IP'):
        ns.setparam("base_layer", "dh_dir", '0,0,1')
        ns.setktens("-1x2", "0.5z2")
        ns.setangle("base_layer", 90, 0)
        #ns.dwall("-x", "-y", 200e-9, 0)
        ns.dwall('-x', '-y', 666e-9, 0) #depending on system length
        dp_index = 2
    elif (geometry.strip() == 'OOP'):
        ns.setparam("base_layer", "dh_dir", '1,0,0')
        ns.setktens("-1z2", "0.5x2")
        ns.setangle("base_layer", 0, 0)
        ns.dwall("-z", "x", 200e-9, 0)
        dp_index = 3
    else:
        print('which geometry??')
        stop

    return ns,dp_index

def RunSimulation(geometry,dmi,exMech,V,H,dmivalue,omega,t0,t1,t2,t3,t4,t5):
    ns,dp_index = Init(geometry)
    utils.delete_files('./temp')

    Dinhom = dmivalue
    Dhom = 2e3
    if (dmi == 'both'):
        ns.addmodule("base_layer", "DMexchange")
        ns.setparam("base_layer", "D_AFM", Dinhom)
        ns.setparam("base_layer", "Dh", Dhom)

    elif (dmi == 'onlyBulk'):
        ns.addmodule("base_layer", "DMexchange")
        ns.setparam("base_layer", "D_AFM", Dinhom)
        ns.setparam("base_layer", "Dh", 0)

    elif (dmi == 'onlyHom'):
        ns.addmodule("base_layer", "DMexchange")
        ns.setparam("base_layer", "D_AFM", 0)
        ns.setparam("base_layer", "Dh", Dhom)

    elif (dmi == 'none'):
        ns.setparam("base_layer", "D_AFM", 0)
        ns.setparam("base_layer", "Dh", 0)
        ns.addmodule("base_layer", "exchange")

    elif (dmi == 'interfacial'):
        ns.addmodule("base_layer", "iDMexchange")
        ns.setparam("base_layer", "D_AFM", Dinhom)
        ns.setparam("base_layer", "Dh", Dhom)
    else:
        print('which DMI??')
        stop

    if (exMech=='Torque'):

        ns.addmodule('base_layer', 'SOTfield')
        ns.addmodule('base_layer', 'transport')
        ns.temperature('0.3K')
        ns.setparam('base_layer','SHA','1')
        ns.setparam('base_layer','flST','1')

        ns.setstage('Relax')
        ns.addstage('V')
        ns.editstagevalue('1',str(-0.001*V))
        ns.addstage('V')
        ns.editstagevalue('2','0')
```

```
    ns.addstage('V')
    ns.editstagevalue('3',str(0.001*V))
    ns.addstage('V')
    ns.editstagevalue('4','0')

    ns.editstagestop(0, 'time', t1*1e-12)
    ns.editstagestop(1, 'time', t2*1e-12)
    ns.editstagestop(2, 'time', t3*1e-12)
    ns.editstagestop(3, 'time', t4*1e-12)
    ns.editstagestop(4, 'time', t5*1e-12)

    ns.setode('sLLG','RK4')
    ns.setdt(1e-15)

    ns.addelectrode('0,0,0,500e-9,0,4e-9') #electrodes set virtual current direction
    ns.addelectrode('0,20e-9,0,500e-9,20e-9,4e-9')
    ns.designateground('1') #set the ground electrode

    if (geometry=='OOP'):
        ns.setparam('STp','1,0,0')
    ns.setparamvar('SHA','equation','step(x-50e-9)-step(x-70e-9)')
    ns.setparamvar('flST','equation','step(x-50e-9)-step(x-70e-9)')

elif (exMech == 'Bfield'):
    ns.setstage("Relax")
    ns.addstage("Relax")
    ns.addstage("Hequation")
    ns.addstage("Relax")
    ns.addstage("Hequation")
    ns.addstage("Relax")

    #ns.temperature('0.1K')

    ns.editstagestop(0, "time", t0*1e-12) # 1. relax
    ns.editstagestop(1, "time", t1*1e-12) # 1. relax
    ns.editstagestop(2, "time", t2*1e-12) # 1. excite
    ns.editstagestop(3, "time", t3*1e-12) # 2. relax
    ns.editstagestop(4, "time", t4*1e-12) # 2. excite
    ns.editstagestop(5, "time", t5*1e-12) # 3. relax

    if (geometry == 'IP'):
        ns.editstagevalue(index = "2", value='0, H0*cos(w*t)*step(s-x), H0*sin(w*t)*step(s-x)')
        ns.editstagevalue(index = "4", value='0, H0*sin(w*t)*step(s-x), H0*cos(w*t)*step(s-x)')
    elif (geometry == 'OOP'):
        ns.editstagevalue(index = "2", value='H0*cos(w*t)*step(s-x), H0*sin(w*t)*step(s-x),0')
        ns.editstagevalue(index = "4", value='H0*sin(w*t)*step(s-x), H0*cos(w*t)*step(s-x),0')
    else:
        print('which geo?')
        stop
    ns.setode('LLG','RK4')
    ns.setdt(2e-15)
    #if temperature > 0 :
    #ns.setode('sLLG','RK4')
    #ns.setdt(1e-15)
    ns.equationconstants("H0", H)
    ns.equationconstants("w", omega)
    ns.equationconstants("s", 2e-8) #injector width


else:
    print('dont know how to excite')
    stop
ns.cuda(1)
ns.setdata("commbuf")
ns.adddata("time")
savedt = 0.1e-12
for i in range(0, 6):
    ns.editdatasave(i, "time", savedt)
ns.dp_getexactprofile(start = "0e-9, 10e-9, 0e-9", end = "500e-9, 10e-9, 0", step =  "4e-9", dp_index= "0",
bufferCommand=True)
```

```
    ns.dp_save("filepath/DW_pos_%iter%.txt", dp_indexes=dp_index, bufferCommand=True)
    # index 1 = x, index 2 = y, index 3 = z component, used to read out the DW position.
    # Raw data is only saved in a temp/ directory for the analysis, only processed data is saved.

    ns.Run()

def RunSpikes(geometry,dmi,exMech,H,dmivalue,omega,t0,t1,t2,t3,t4,t5,t6):
    ns,dp_index = Init(geometry)
    utils.delete_files('./temp')
    Dinhom = dmivalue
    Dhom = 2e3
    if (dmi == 'both'):
        ns.addmodule("base_layer", "DMexchange")
        ns.setparam("base_layer", "D_AFM", Dinhom)
        ns.setparam("base_layer", "Dh", Dhom)

    elif (dmi == 'onlyBulk'):
        ns.addmodule("base_layer", "DMexchange")
        ns.setparam("base_layer", "D_AFM", Dinhom)
        ns.setparam("base_layer", "Dh", 0)

    elif (dmi == 'onlyHom'):
        ns.addmodule("base_layer", "DMexchange")
        ns.setparam("base_layer", "D_AFM", 0)
        ns.setparam("base_layer", "Dh", Dhom)

    elif (dmi == 'none'):
        ns.setparam("base_layer", "D_AFM", 0)
        ns.setparam("base_layer", "Dh", 0)
        ns.addmodule("base_layer", "exchange")

    elif (dmi == 'interfacial'):
        ns.addmodule("base_layer", "iDMexchange")
        ns.setparam("base_layer", "D_AFM", Dinhom)
        ns.setparam("base_layer", "Dh", Dhom)

    if (exMech == 'Bfield'):
        ns.setstage("Relax")
        ns.addstage("Hequation")
        ns.addstage("Relax")
        ns.addstage("Hequation")
        ns.addstage("Relax")
        ns.addstage("Hequation")
        ns.addstage("Relax")

        ns.editstagestop(0, "time", t0*1e-12) # 1. relax
        ns.editstagestop(1, "time", t1*1e-12) #pull
        ns.editstagestop(2, "time", t2*1e-12) #relax
        ns.editstagestop(3, "time", t3*1e-12) #pull
        ns.editstagestop(4, "time", t4*1e-12) #relax
        ns.editstagestop(5, "time", t5*1e-12) #pull
        ns.editstagestop(6, "time", t6*1e-12) #relax

        if (geometry == 'IP'):
            ns.editstagevalue(index = "1", value='0, H0*cos(w*t)*step(s-x), H0*sin(w*t)*step(s-x)')
            ns.editstagevalue(index = "3", value='0, H0*cos(w*t)*step(s-x), H0*sin(w*t)*step(s-x)')
            ns.editstagevalue(index = "5", value='0, H0*cos(w*t)*step(s-x), H0*sin(w*t)*step(s-x)')
        elif (geometry == 'OOP'):
            ns.editstagevalue(index = "2", value='H0*cos(w*t)*step(s-x), H0*sin(w*t)*step(s-x),0')
            ns.editstagevalue(index = "4", value='H0*sin(w*t)*step(s-x), H0*cos(w*t)*step(s-x),0')
        else:
            print('which geo?')
            stop
        ns.setode('LLG','RK4')
        ns.setdt(2e-15)
        ns.equationconstants("H0", H)
        ns.equationconstants("w", omega )
        ns.equationconstants("s", 2e-8)
```

```
    else:
        print('dont know how to excite')
        stop
    ns.cuda(1)
    ns.setdata("commbuf")
    ns.adddata("time")
    savedt = 0.1e-12
    for i in range(0, 7):
        ns.editdatasave(i, "time", savedt)
    ns.dp_getexactprofile(start = "0e-9, 10e-9, 0e-9", end = "500e-9, 10e-9, 0", step =  "4e-9", dp_index= "0",
    bufferCommand=True)
    ns.dp_save("temp/Spikes-DW_pos_%iter%.txt", dp_indexes=dp_index, bufferCommand=True)

    ns.Run()

def RunSpikesPumpReadout(geometry,dmi,Dinhom):
    H = [2.5e7]
    omega = 6.25e13
    t0,t1,t2,t3,t4,t5,t6 = 25, 10, 20, 4, 2, 4 , 25 # times depend on material parameters and anisotropy slope
    outputfile = 'filepath/signal.txt' # overall signal

    # background, will be subtracted. From system with pumped magnons but no DW present.
    # In order to do that, comment out the initialization of the DW

    outputfileBackground = 'filepath/backround.txt'

    ns,dp_index = Init(geometry)

    utils.delete_files('./temp')

    Dhom = 2e3
    if (dmi == 'both'):
        ns.addmodule("base_layer", "DMexchange")
        ns.setparam("base_layer", "D_AFM", Dinhom)
        ns.setparam("base_layer", "Dh", Dhom)

    elif (dmi == 'onlyBulk'):
        ns.addmodule("base_layer", "DMexchange")
        ns.setparam("base_layer", "D_AFM", Dinhom)
        ns.setparam("base_layer", "Dh", 0)

    elif (dmi == 'onlyHom'):
        ns.addmodule("base_layer", "DMexchange")
        ns.setparam("base_layer", "D_AFM", 0)
        ns.setparam("base_layer", "Dh", Dhom)

    elif (dmi == 'none'):
        ns.setparam("base_layer", "D_AFM", 0)
        ns.setparam("base_layer", "Dh", 0)
        ns.addmodule("base_layer", "exchange")

    elif (dmi == 'interfacial'):
        ns.addmodule("base_layer", "iDMexchange")
        ns.setparam("base_layer", "D_AFM", Dinhom)
        ns.setparam("base_layer", "Dh", Dhom)


    ns.setstage("Relax")
    ns.addstage("Hequation")
    ns.addstage("Relax")
    ns.addstage("Hequation")
    ns.addstage("Relax")
    ns.addstage("Hequation")
    ns.addstage("Relax")

    ns.editstagestop(0, "time", t0*1e-12) # 1. relax
    ns.editstagestop(1, "time", t1*1e-12) #pull
    ns.editstagestop(2, "time", t2*1e-12) #relax
    ns.editstagestop(3, "time", t3*1e-12) #pull
    ns.editstagestop(4, "time", t4*1e-12) #relax
```

```
ns.editstagestop(5, "time", t5*1e-12) #pull
ns.editstagestop(6, "time", t6*1e-12) #relax

if (geometry == 'IP'):
    ns.editstagevalue(index = "1", value='0, H0*cos(w*t)*step(s-x), H0*sin(w*t)*step(s-x)')
    ns.editstagevalue(index = "3", value='0, H0*cos(w*t)*step(s-x), H0*sin(w*t)*step(s-x)')
    ns.editstagevalue(index = "5", value='0, H0*cos(w*t)*step(s-x), H0*sin(w*t)*step(s-x)')
#elif (geometry == 'OOP'):
    # was not used, but analogous
else:
    print('which geo?')
    stop
ns.setode('LLG','RK4')
ns.setdt(2e-15)
ns.equationconstants("H0", H)
ns.equationconstants("w", omega)
ns.equationconstants("s", 2e-8)


ns.cuda(1)
ns.setdata("commbuf")
ns.adddata("time")
savedt = 0.1e-12
for i in range(0,7):
    ns.editdatasave(i, "time", savedt)
ns.dp_getexactprofile(start = "0e-9, 10e-9, 0e-9", end = "500e-9, 10e-9, 0", step =  "4e-9", dp_index= "0",
bufferCommand=True)
ns.dp_save("temp/Spikes-DW_pos_%iter%.txt", dp_indexes=dp_index, bufferCommand=True)
x1 = 250
#x2 = 400 #if you want to read out at the right, too ('detector 2')
#crossterms detecor 1

ns.adddata('<mxdmdt>',[(x1-10)*1e-9, 2.0e-9, 2.0e-9, (x1+10)*1e-9, 18.0e-9, 2.0e-9])
ns.adddata('<mxdmdt2>',[(x1-10)*1e-9, 2e-9, 2e-9, (x1+10)*1e-9, 18e-9, 2e-9])
ns.adddata('<m2xdmdt>',[(x1-10)*1e-9, 2.0e-9, 2.0e-9, (x1+10)*1e-9, 18.0e-9, 2.0e-9])
ns.adddata('<mxdm2dt>',[(x1-10)*1e-9, 2e-9, 2e-9, (x1+10)*1e-9, 18e-9, 2e-9])


#crossterms detecor 2
#ns.adddata('<mxdmdt>',[406.0e-9, 2.0e-9, 2.0e-9, 406.0e-9, 18.0e-9, 2.0e-9])
#ns.adddata('<mxdmdt2>',[406.0e-9, 2e-9, 2e-9, 406.0e-9, 18e-9, 2e-9])
#ns.adddata('<m2xdmdt>',[406.0e-9, 2.0e-9, 2.0e-9, 406.0e-9, 18.0e-9, 2.0e-9])
#ns.adddata('<mxdm2dt>',[406.0e-9, 2e-9, 2e-9, 406.0e-9, 18e-9, 2e-9])

# dmdt detector 1
ns.adddata('<dmdt>',[(x1-10)*1e-9, 2.0e-9, 2.0e-9, (x1+10)*1e-9, 18.0e-9, 2.0e-9])
ns.adddata('<dmdt2>',[(x1-10)*1e-9, 2.0e-9, 2.0e-9, (x1+10)*1e-9, 18.0e-9, 2.0e-9])

#crossterms detecor 2
#ns.adddata('<dmdt>',[406.0e-9, 2.0e-9, 2.0e-9, 406.0e-9, 18.0e-9, 2.0e-9])
#ns.adddata('<dmdt2>',[406.0e-9, 2.0e-9, 2.0e-9, 406.0e-9, 18.0e-9, 2.0e-9])

ns.savedatafile(outputfile) # or outputfileBackground

ns.Run()


dS = np.array(pd.read_csv(outputfile, sep = '\s+', header = None, index_col = False,skiprows=9))
dB = np.array(pd.read_csv(outputfileBackground, sep = '\s+', header = None, index_col = False,skiprows=9))
time = dS[:,3]

# 'signal' must be scaled with real part of spin mixing conductance. Just from first detector
signal1N = np.subtract(np.add(dS[:,4],dS[:,7]),np.add(dS[:,10],dS[:,13]))
background1N = np.subtract(np.add(dB[:,4],dB[:,7]),np.add(dB[:,10],dB[:,13]))
cleanSignal1N = np.subtract(signal1N,background1N)

# if you want to read out the magnetization, not Neel, add instead of subtract the sublattice contributions
signal1M = np.add(np.add(dS[:,4],dS[:,7]),np.add(dS[:,10],dS[:,13]))
background1M = np.add(np.add(dB[:,4],dB[:,7]),np.add(dB[:,10],dB[:,13]))
cleanSignal1M = np.subtract(signal1N,background1N)
```

```
    # 'ImagSig' is term after imaginary part.
    ImagSig = np.add(dS[:,16],dS[:,19])
    backroundImagSig = np.add(dB[:,16],dB[:,19])
    cleanImagSig = np.subtract(ImagSig,backroundImagSig)

    #signal2 = np.subtract(np.add(dS[:,16],dS[:,19]),np.add(dS[:,22],dS[:,25]))
    #background2 = np.subtract(np.add(dB[:,16],dB[:,19]),np.add(dB[:,22],dB[:,25]))
    #cleanSignal2 = np.subtract(signal2,background2)

    plt.plot((time/1e-12)-25,signal1N/1e9,marker='x',c='black',label='signal'))
    #plt.plot((time/1e-12)-25,signal1M/1e9,marker='x',c='gray',label='signal from Magnetization')# )

    plt.plot((time/1e-12)-25,background1N/1e9,marker='x',c='red',label='background')# M')
    #plt.plot((time/1e-12)-25,background1M/1e9,marker='x',c='violet',label='background')# N')

    #plt.scatter((time/1e-12)-25,cleanSignal1M/1e9,marker='x',c='blue',label='clean signal')# M')

    # test if including the imaginary part makes a difference
    Gr = 1
    Gi=Gr
    overallSigFactor1 = Gr*np.add(cleanSignal1N,cleanSignal1M)-Gi*cleanImagSig
    Gi=0
    overallSigFactor0 = Gr*np.add(cleanSignal1N,cleanSignal1M)-Gi*cleanImagSig


    plt.plot((time/1e-12)-25,cleanSignal1N/1e9,c='blue',marker='o',label='clean signal\n'+r'$G_i=G_r$')# N')

    plt.plot((time/1e-12)-25,overallSigFactor0/1e9,linewidth=3,linestyle='dashed',c='green',label=r'$G_i=0$')# N')

    Ttotal= t0+t1+t2+t3+t4+t5+t6
    T2 = t1+t2+t3+t4+t5+t6


    plt.xlabel('t (ps)')
    plt.legend(handlelength=1,handletextpad=0.5)
    plt.subplots_adjust(left=0.2,right=0.95,bottom=0.15,top=0.95)

    plt.xlim(0,25)
    plt.ylabel(r'$\mu_x$ (GHz)')

def get_x_DW_2(path_dw_folder):
    '''imports dw postition at times
    returns times and dw
    '''

    dw = np.array([])

    for file in sort_alphanumeric(os.listdir(path_dw_folder)):
        mx = np.loadtxt(os.path.join(path_dw_folder, file))
        idx = np.abs(mx).argmin()
        # either use maximum or minimum of observable of choice.
        # observable is set in the init function (dp_index): choose x, y or z component of the magnetization
        dw_pos = 4e-9 * idx - 2e-9 #4e-9 comes from lattice constant
        dw = np.append(dw, dw_pos)
    return dw


def AnalyzeData(rawdata,Savefilename,totalT): #reads out the DW position from the raw data through
    dw = get_x_DW_2(rawdata)
    times = np.linspace(0,totalT,len(dw))
    results = np.zeros((2,int(len(dw))))
    results[0,:] = times
    results[1,:] = dw
    np.savetxt(Savefilename,results)
    return times,dw



def SimulateWithTorque(geos,DMIS,bulkDMIvalues,N,Vs):
```

```python
# times are just example
t0,t1,t2,t3,t4,t5 = 35,5,25,40,25,40
totalT = t0+t1+t2+t3+t4+t5
def RunSim(geometry,dmi,V,dmivalue,t0,t1,t2,t3,t4,t5):
    ns,dp_index = Init(geometry)
    utils.delete_files('./temp')

    Dinhom = dmivalue
    Dhom = 2e3
    if (dmi == 'both'):
        ns.addmodule("base_layer", "DMexchange")
        ns.setparam("base_layer", "D_AFM", Dinhom)
        ns.setparam("base_layer", "Dh", Dhom)

    elif (dmi == 'onlyBulk'):
        ns.addmodule("base_layer", "DMexchange")
        ns.setparam("base_layer", "D_AFM", Dinhom)
        ns.setparam("base_layer", "Dh", 0)

    elif (dmi == 'onlyHom'):
        ns.addmodule("base_layer", "DMexchange")
        ns.setparam("base_layer", "D_AFM", 0)
        ns.setparam("base_layer", "Dh", Dhom)

    elif (dmi == 'none'):
        ns.setparam("base_layer", "D_AFM", 0)
        ns.setparam("base_layer", "Dh", 0)
        ns.addmodule("base_layer", "exchange")

    elif (dmi == 'interfacial'):
        ns.addmodule("base_layer", "iDMexchange")
        ns.setparam("base_layer", "D_AFM", Dinhom)
        ns.setparam("base_layer", "Dh", Dhom)



    ns.addmodule('base_layer', 'SOTfield')
    ns.addmodule('base_layer', 'transport')
    ns.temperature('0.3K')
    ns.setparam('base_layer','SHA','1')
    ns.setparam('base_layer','flST','1')

    ns.setstage('Relax')
    ns.addstage('V')
    ns.editstagevalue('1',str(-0.001*V))
    ns.addstage('V')
    ns.editstagevalue('2','0')
    ns.addstage('V')
    ns.editstagevalue('3',str(0.001*V))
    ns.addstage('V')
    ns.editstagevalue('4','0')

    ns.editstagestop(0, 'time', (t0+t1)*1e-12)
    ns.editstagestop(1, 'time', t2*1e-12)
    ns.editstagestop(2, 'time', t3*1e-12)
    ns.editstagestop(3, 'time', t4*1e-12)
    ns.editstagestop(4, 'time', t5*1e-12)

    ns.setode('sLLG','RK4')
    ns.setdt(1e-15)

    ns.addelectrode('0,0,0,500e-9,0,4e-9') #electrodes set virtual current direction
    ns.addelectrode('0,20e-9,0,500e-9,20e-9,4e-9')
    ns.designateground('1') #set the ground electrode

    if (geometry=='OOP'):
        ns.setparam('STp','1,0,0')
    ns.setparamvar('SHA','equation','step(x-50e-9)-step(x-70e-9)')
    ns.setparamvar('flST','equation','step(x-50e-9)-step(x-70e-9)')
```

```
        ns.cuda(1)
        ns.setdata("commbuf")
        ns.adddata("time")
        savedt = 0.1e-12
        for i in range(0, 6):
            ns.editdatasave(i, "time", savedt)
        ns.dp_getexactprofile(start = "0e-9, 10e-9, 0e-9", end = "500e-9, 10e-9, 0", step =  "4e-9", dp_index= "0",
        bufferCommand=True)
        ns.dp_save("temp/TorqueEx-T=0p3-DW_pos_%iter%.txt", dp_indexes=dp_index, bufferCommand=True)

        ns.Run()

    for r in range(N):
        for v in range(len(bulkDMIvalues)):
            bulkDMIvalue = bulkDMIvalues[v]
            for geometry in geos:
                for dmi in range(len(DMIS)):
                    Voltagevalue = Vs[dmi]
                    RunSim(geometry.strip(),DMIS[dmi],Voltagevalue,bulkDMIvalue*1e-6,t0,t1,t2,t3,t4,t5)
                    Savefilename = 'filepath/'+str(geometry)+'-'+str(DMIS[dmi])+'-run'+str(r+10)+'-DMI='+str(bulkDMIvalue)
                    +'uJperm2-V='+str(Voltagevalue)
                    times,dw = AnalyzeData('temp/',Savefilename+'.txt',totalT)
                    #plt.scatter(times,dw)
                    #plt.show()
    return Vs

def SimulatePumpWithB_IP(geos,DMIS,bulkDMIvalues,N):
    # the following numbers are just examples
    H = [2.5e7]
    omega = 6.25e13
    t0,t1,t2,t3,t4,t5 =  0,25,25,25,25,20 # for B ex
    totalT = t0+t1+t2+t3+t4+t5
    #t0,t1,t2,t3,t4,t5,t6 = 25, 10, 2, 10, 2 ,10 , 25 #for spikes
    outputfile = 'filepath/Data/testPump.txt'
    outputfileBackground = 'filepath/Data/testPump-Background.txt'

    def RunSimH(geometry,dmi,dmivalue):
        Dinhom = dmivalue
        Dhom = 2e3

        ns,dp_index = Init(geometry)
        utils.delete_files('./temp')

        Dinhom = dmivalue
        Dhom = 2e3
        if (dmi == 'both'):
            ns.addmodule("base_layer", "DMexchange")
            ns.setparam("base_layer", "D_AFM", Dinhom)
            ns.setparam("base_layer", "Dh", Dhom)

        elif (dmi == 'onlyBulk'):
            ns.addmodule("base_layer", "DMexchange")
            ns.setparam("base_layer", "D_AFM", Dinhom)
            ns.setparam("base_layer", "Dh", 0)

        elif (dmi == 'onlyHom'):
            ns.addmodule("base_layer", "DMexchange")
            ns.setparam("base_layer", "D_AFM", 0)
            ns.setparam("base_layer", "Dh", Dhom)

        elif (dmi == 'none'):
            ns.setparam("base_layer", "D_AFM", 0)
            ns.setparam("base_layer", "Dh", 0)
            ns.addmodule("base_layer", "exchange")

        elif (dmi == 'interfacial'):
            ns.addmodule("base_layer", "iDMexchange")
            ns.setparam("base_layer", "D_AFM", Dinhom)
            ns.setparam("base_layer", "Dh", Dhom)
```

```
    ns.setstage("Relax")
    ns.addstage("Relax")
    ns.addstage("Hequation")
    ns.addstage("Relax")
    ns.addstage("Hequation")
    ns.addstage("Relax")

    ns.editstagestop(0, "time", t0*1e-12) # 1. relax
    ns.editstagestop(1, "time", t1*1e-12) # 1. relax
    ns.editstagestop(2, "time", t2*1e-12) # 1. spike
    ns.editstagestop(3, "time", t3*1e-12) # 2. relax
    ns.editstagestop(4, "time", t4*1e-12) # 2. spike
    ns.editstagestop(5, "time", t5*1e-12) # 3. relax

    if (geometry == 'IP'):
        ns.editstagevalue(index = "2", value='0, H0*cos(w*t)*step(s-x), H0*sin(w*t)*step(s-x)')
        ns.editstagevalue(index = "4", value='0, H0*sin(w*t)*step(s-x), H0*cos(w*t)*step(s-x)')

    elif (geometry == 'OOP'):
        ns.editstagevalue(index = "2", value='H0*cos(w*t)*step(s-x), H0*sin(w*t)*step(s-x),0')
        ns.editstagevalue(index = "4", value='H0*sin(w*t)*step(s-x), H0*cos(w*t)*step(s-x),0')
    else:
        print('which geo?')
        stop
    ns.setode('LLG','RK4')
    ns.setdt(2e-15)
    ns.equationconstants("H0", H)
    ns.equationconstants("w", omega )
    ns.equationconstants("s", 2e-8)

    ns.cuda(1)

    savedt = 0.1e-12
    for i in range(0, 6):
        ns.editdatasave(i, "time", savedt)
    ns.adddata('<mxdmdt>',[x1e-9, 2.0e-9, 2.0e-9, x1e-9, 18.0e-9, 2.0e-9]) #detecor - 63nm away
    ns.adddata('<mxdmdt2>',[x1e-9, 2e-9, 2e-9, x1e-9, 18e-9, 2e-9])
    ns.adddata('<m2xdmdt>',[x1e-9, 2.0e-9, 2.0e-9, x1e-9, 18.0e-9, 2.0e-9])
    ns.adddata('<mxdm2dt>',[x1e-9, 2e-9, 2e-9, x1e-9, 18e-9, 2e-9])

    ns.adddata('<mxdmdt>',[406.0e-9, 2.0e-9, 2.0e-9, 406.0e-9, 18.0e-9, 2.0e-9]) #detecor + 63nm away
    ns.adddata('<mxdmdt2>',[406.0e-9, 2e-9, 2e-9, 406.0e-9, 18e-9, 2e-9])
    ns.adddata('<m2xdmdt>',[406.0e-9, 2.0e-9, 2.0e-9, 406.0e-9, 18.0e-9, 2.0e-9]) #detecor + 63nm away
    ns.adddata('<mxdm2dt>',[406.0e-9, 2e-9, 2e-9, 406.0e-9, 18e-9, 2e-9])

    ns.savedatafile(outputfile)
    #ns.setdata("commbuf")
    #ns.dp_getexactprofile(start = "0e-9, 10e-9, 0e-9", end = "500e-9, 10e-9, 0", step =  "4e-9", dp_index= "0",
    bufferCommand=True)
    #ns.dp_save("temp/BfieldEx-DW_pos_%iter%.txt", dp_indexes=dp_index, bufferCommand=True)
    ns.Run()


dS = np.array(pd.read_csv(outputfile, sep = '\s+', header = None, index_col = False,skiprows=9))
dB = np.array(pd.read_csv(outputfileBackground, sep = '\s+', header = None, index_col = False,skiprows=9))
signal1 = np.subtract(np.add(dS[:,4],dS[:,7]),np.add(dS[:,10],dS[:,13]))
background1 = np.subtract(np.add(dB[:,4],dB[:,7]),np.add(dB[:,10],dB[:,13]))
cleanSignal1 = np.subtract(signal1,background1)
signal2 = np.subtract(np.add(dS[:,16],dS[:,19]),np.add(dS[:,22],dS[:,25]))
background2 = np.subtract(np.add(dB[:,16],dB[:,19]),np.add(dB[:,22],dB[:,25]))
cleanSignal2 = np.subtract(signal2,background2)

fig, ax1 = plt.subplots()

T2 = t2+t3+t4+t5

ax2 = ax1.twinx()
Savefilename = 'filepath/DMI='+str(bulkDMIvalues[0])+'uJperm2'
```

```
    times = np.loadtxt(Savefilename+'.txt')[0]
    dw = np.loadtxt(Savefilename+'.txt')[1]
    ax2.axhspan(0,20,0,25/T2,color='orange')#,label='injector')
    ax2.axhspan(0,20,50/T2,75/T2,color='orange')
    ax2.set_xlim(0,totalT-25)
    ax2.plot([3],[9],marker=r'$\circlearrowleft$',ms=12,color='black')
    ax2.plot([13],[9],marker=r'$\circlearrowleft$',ms=12,color='black')
    ax2.plot([23],[9],marker=r'$\circlearrowleft$',ms=12,color='black')
    ax2.plot([53],[9],marker=r'$\circlearrowright$',ms=12,color='black')
    ax2.plot([63],[9],marker=r'$\circlearrowright$',ms=12,color='black')
    ax2.plot([73],[9],marker=r'$\circlearrowright$',ms=12,color='black')
    ax2.set_ylim(0,430)
    ax2.set_ylabel(r'x$_\mathrm{DW}$ [nm]')
    ax2.set_xlabel('t [ps]')
    ax2.plot(times-25,dw/1e-9,color='black',linewidth=3)

    ax1.plot(times-25,cleanSignal1/1e9,label=r'$\mu_x$(d$_1$)',color='royalblue',linewidth=3)
    ax1.plot(times-25,cleanSignal2/1e9,label=r'$\mu_x$(d$_2$)',color='navy',linewidth=3)


    ax2.hlines(333,0,T2,colors='gray',linestyles='dashed',label=r'$x_0$')
    ax2.hlines(406,0,T2,colors='gray',linestyles='dotted',label=r'd$_1$')
    ax2.hlines(260,0,T2,colors='gray',linestyles='dotted',label=r'd$_2$')
    ax2.text(85,270,r'(d$_1$)',size=15,color='royalblue')
    ax2.text(85,380,r'(d$_2$)',size=15,color='navy')
    ax2.text(1.5,340,r'x$_0$',size=15,color='gray')

    ax1.set_ylabel(r'x$_\mathrm{DW}$ [nm]',color='blue')
    ax1.set_ylabel(r'$\mu_x$ [1/ps]')
    ax1.set_xlabel('t [ps]')
    ax1.tick_params(axis='y', colors='blue')
    plt.subplots_adjust(left=0.2,right=0.8,bottom=0.15,top=0.95)

    ax1.legend(handlelength=0.5,handletextpad=0.5)
    #ax2.legend()
    plt.show()

def SimulatePumpWithB_OOP(geos,DMIS,bulkDMIvalues,N):
    H = [4e7]
    omega = 6.25e13
    t0,t1,t2,t3,t4,t5 =  0,25,25,25,25,20 # for B ex
    totalT = t0+t1+t2+t3+t4+t5
    #t0,t1,t2,t3,t4,t5,t6 = 25, 10, 2, 10, 2 ,10 , 25 #for spikes
    x1 = 293.0e-9 #position of detectors, variable
    x2 = 373.0e-9

    outputfile = 'filepath/signal.txt'
    outputfileBackground = 'filepath/background.txt' #see above

    def RunSimH(geometry,dmi,dmivalue):
        Dinhom = dmivalue
        Dhom = 2e3

        ns,dp_index = Init(geometry)
        utils.delete_files('./temp')

        Dinhom = dmivalue
        Dhom = 2e3
        if (dmi == 'both'):
            ns.addmodule("base_layer", "DMexchange")
            ns.setparam("base_layer", "D_AFM", Dinhom)
            ns.setparam("base_layer", "Dh", Dhom)

        elif (dmi == 'onlyBulk'):
            ns.addmodule("base_layer", "DMexchange")
            ns.setparam("base_layer", "D_AFM", Dinhom)
            ns.setparam("base_layer", "Dh", 0)

        elif (dmi == 'onlyHom'):
            ns.addmodule("base_layer", "DMexchange")
```

```
        ns.setparam("base_layer", "D_AFM", 0)
        ns.setparam("base_layer", "Dh", Dhom)

    elif (dmi == 'none'):
        ns.setparam("base_layer", "D_AFM", 0)
        ns.setparam("base_layer", "Dh", 0)
        ns.addmodule("base_layer", "exchange")

    elif (dmi == 'interfacial'):
        ns.addmodule("base_layer", "iDMexchange")
        ns.setparam("base_layer", "D_AFM", Dinhom)
        ns.setparam("base_layer", "Dh", Dhom)


    ns.setstage("Relax")
    ns.addstage("Relax")
    ns.addstage("Hequation")
    ns.addstage("Relax")
    ns.addstage("Hequation")
    ns.addstage("Relax")

    ns.editstagestop(0, "time", t0*1e-12) # 1. relax
    ns.editstagestop(1, "time", t1*1e-12) # 1. relax
    ns.editstagestop(2, "time", t2*1e-12) # 1. spike
    ns.editstagestop(3, "time", t3*1e-12) # 2. relax
    ns.editstagestop(4, "time", t4*1e-12) # 2. spike
    ns.editstagestop(5, "time", t5*1e-12) # 3. relax

    if (geometry == 'IP'):
        ns.editstagevalue(index = "2", value='0, H0*cos(w*t)*step(s-x), H0*sin(w*t)*step(s-x)')
        ns.editstagevalue(index = "4", value='0, H0*sin(w*t)*step(s-x), H0*cos(w*t)*step(s-x)')

    elif (geometry == 'OOP'):
        ns.editstagevalue(index = "2", value='H0*cos(w*t)*step(s-x), H0*sin(w*t)*step(s-x),0')
        ns.editstagevalue(index = "4", value='H0*sin(w*t)*step(s-x), H0*cos(w*t)*step(s-x),0')
    else:
        print('which geo?')
        stop
    ns.setode('LLG','RK4')
    ns.setdt(2e-15)
    ns.equationconstants("H0", H)
    ns.equationconstants("w", omega )
    ns.equationconstants("s", 2e-8)

    ns.cuda(1)

    savedt = 0.1e-12
    for i in range(0, 6):
        ns.editdatasave(i, "time", savedt)

    ns.adddata('<mxdmdt>',[x1, 2.0e-9, 2.0e-9, x1, 18.0e-9, 2.0e-9]) #detecor - 63nm away
    ns.adddata('<mxdmdt2>',[x1, 2.0e-9, 2.0e-9, x1, 18e-9, 2e-9])
    ns.adddata('<m2xdmdt>',[x1, 2.0e-9, 2.0e-9, x1, 18.0e-9, 2.0e-9])
    ns.adddata('<mxdm2dt>',[x1, 2.0e-9, 2.0e-9, x1, 18.0e-9, 2.0e-9])

    ns.adddata('<mxdmdt>',[x2, 2.0e-9, 2.0e-9, x2, 18.0e-9, 2.0e-9]) #detecor - 63nm away
    ns.adddata('<mxdmdt2>',[x2, 2.0e-9, 2.0e-9, x2, 18e-9, 2e-9])
    ns.adddata('<m2xdmdt>',[x2, 2.0e-9, 2.0e-9, x2, 18.0e-9, 2.0e-9])
    ns.adddata('<mxdm2dt>',[x2, 2.0e-9, 2.0e-9, x2, 18.0e-9, 2.0e-9])

    ns.savedatafile(outputfileBackground)
    ns.Run()


dS = np.array(pd.read_csv(outputfile, sep = '\s+', header = None, index_col = False,skiprows=9))
dB = np.array(pd.read_csv(outputfileBackground, sep = '\s+', header = None, index_col = False,skiprows=9))

signal1 = np.subtract(np.add(dS[:,6],dS[:,9]),np.add(dS[:,12],dS[:,15]))
background1 = np.subtract(np.add(dB[:,6],dB[:,9]),np.add(dB[:,12],dB[:,15]))
cleanSignal1 = np.subtract(signal1,background1)
```

```
    signal2 = np.subtract(np.add(dS[:,18],dS[:,21]),np.add(dS[:,25],dS[:,27]))
    background2 = np.subtract(np.add(dB[:,18],dB[:,21]),np.add(dB[:,25],dB[:,27]))
    cleanSignal2 = np.subtract(signal2,background2)


    fig, ax1 = plt.subplots()


    #t = data[3]



    T2 = t2+t3+t4+t5

    ax2 = ax1.twinx()
    Savefilename = 'example/DMI='+str(bulkDMIvalues[0])+'uJperm2'
    times = np.loadtxt(Savefilename+'.txt')[0]
    dw = np.loadtxt(Savefilename+'.txt')[1]
    ax2.axhspan(0,20,0,25/T2,color='orange')#,label='injector')
    ax2.axhspan(0,20,50/T2,75/T2,color='orange')
    ax2.set_xlim(0,totalT-25)
    ax2.plot([3],[9],marker=r'$\circlearrowleft$',ms=12,color='black')
    ax2.plot([13],[9],marker=r'$\circlearrowleft$',ms=12,color='black')
    ax2.plot([23],[9],marker=r'$\circlearrowleft$',ms=12,color='black')
    ax2.plot([53],[9],marker=r'$\circlearrowright$',ms=12,color='black')
    ax2.plot([63],[9],marker=r'$\circlearrowright$',ms=12,color='black')
    ax2.plot([73],[9],marker=r'$\circlearrowright$',ms=12,color='black')
    ax2.set_ylim(0,430)
    ax2.set_ylabel(r'x$_\mathrm{DW}$ [nm]')
    ax2.set_xlabel('t [ps]')
    ax2.plot(times-25,dw/1e-9,color='black',linewidth=3)

    ax1.plot(times-25,signal1/1e9,label=r'$\mu_x$(d$_1$)',color='royalblue',linewidth=3)


    ax1.plot(times-25,signal2/1e9,label=r'sig2',color='orange',linewidth=3)
    ax1.plot(times-25,background2/1e9,label=r'background2',color='red',linewidth=3)
    ax1.plot(times-25,cleanSignal2/1e9,label=r'$\mu_x$(d$_2$)',color='navy',linewidth=3)
    #ax1.scatter(times-25,nxdndtY,label='muY')
    #ax1.scatter(times-25,nxdndtZ,label='muZ')

    ax2.hlines(333,0,T2,colors='gray',linestyles='dashed',label=r'$x_0$')#)'equilibrium \n position')
    ax2.hlines(x1*1e9,0,T2,colors='gray',linestyles='dotted',label=r'd$_1$')#)'equilibrium \n position')
    ax2.hlines(x2*1e9,0,T2,colors='gray',linestyles='dotted',label=r'd$_2$')#)'equilibrium \n position')
    ax2.text(85,270,r'(d$_1$)',size=15,color='royalblue')
    ax2.text(85,380,r'(d$_2$)',size=15,color='navy')
    ax2.text(1.5,340,r'x$_0$',size=15,color='gray')

    ax1.set_ylabel(r'x$_\mathrm{DW}$ [nm]',color='blue')
    ax1.set_ylabel(r'$\mu_x$ [1/ps]')
    ax1.set_xlabel('t [ps]')
    ax1.tick_params(axis='y', colors='blue')
    plt.subplots_adjust(left=0.2,right=0.8,bottom=0.15,top=0.95)

    ax1.legend(handlelength=0.5,handletextpad=0.5)
    #ax2.legend()
    plt.show()

def SimulateWithBfield(geos,DMIS,bulkDMIvalues,N):
    # the following values are just examples
    H = [4e7]
    omega = 6.25e13
    t0,t1,t2,t3,t4,t5 =  0,40,25,40,25,40 # for B ex
    totalT = t0+t1+t2+t3+t4+t5
    #t0,t1,t2,t3,t4,t5,t6 = 25, 10, 2, 10, 2 ,10 , 25 #for spikes

    def RunSimH(geometry,dmi,dmivalue):
        ns,dp_index = Init(geometry)
        utils.delete_files('./temp')
```

```
Dinhom = dmivalue
Dhom = 2e3
if (dmi == 'both'):
    ns.addmodule("base_layer", "DMexchange")
    ns.setparam("base_layer", "D_AFM", Dinhom)
    ns.setparam("base_layer", "Dh", Dhom)

elif (dmi == 'onlyBulk'):
    ns.addmodule("base_layer", "DMexchange")
    ns.setparam("base_layer", "D_AFM", Dinhom)
    ns.setparam("base_layer", "Dh", 0)

elif (dmi == 'onlyHom'):
    ns.addmodule("base_layer", "DMexchange")
    ns.setparam("base_layer", "D_AFM", 0)
    ns.setparam("base_layer", "Dh", Dhom)

elif (dmi == 'none'):
    ns.setparam("base_layer", "D_AFM", 0)
    ns.setparam("base_layer", "Dh", 0)
    ns.addmodule("base_layer", "exchange")

elif (dmi == 'interfacial'):
    ns.addmodule("base_layer", "iDMexchange")
    ns.setparam("base_layer", "D_AFM", Dinhom)
    ns.setparam("base_layer", "Dh", Dhom)


ns.setstage("Relax")
ns.addstage("Relax")
ns.addstage("Hequation")
ns.addstage("Relax")
ns.addstage("Hequation")
ns.addstage("Relax")

ns.editstagestop(0, "time", t0*1e-12) # 1. relax
ns.editstagestop(1, "time", t1*1e-12) # 1. relax
ns.editstagestop(2, "time", t2*1e-12) # 1. spike
ns.editstagestop(3, "time", t3*1e-12) # 2. relax
ns.editstagestop(4, "time", t4*1e-12) # 2. spike
ns.editstagestop(5, "time", t5*1e-12) # 3. relax

if (geometry == 'IP'):
    ns.editstagevalue(index = "2", value='0, H0*cos(w*t)*step(s-x), H0*sin(w*t)*step(s-x)')
    ns.editstagevalue(index = "4", value='0, H0*sin(w*t)*step(s-x), H0*cos(w*t)*step(s-x)')
    #ns.editstagevalue(index = "2", value='0, 0, H0*sin(w*t)*step(s-x)') # if you wanna test linear polarization
    #ns.editstagevalue(index = "4", value='0, H0*sin(w*t)*step(s-x), 0')

elif (geometry == 'OOP'):
    ns.editstagevalue(index = "2", value='H0*cos(w*t)*step(s-x), H0*sin(w*t)*step(s-x),0')
    ns.editstagevalue(index = "4", value='H0*sin(w*t)*step(s-x), H0*cos(w*t)*step(s-x),0')
else:
    print('which geo?')
    stop
ns.setode('LLG','RK4')
ns.setdt(2e-15)
ns.equationconstants("H0", H)
ns.equationconstants("w", omega )
ns.equationconstants("s", 2e-8)

ns.cuda(1)
#ns.setdata("commbuf")
#ns.adddata("time")
#savedt = 0.1e-12
#for i in range(0, 6):
#    ns.editdatasave(i, "time", savedt)
#ns.dp_getexactprofile(start = "0e-9, 10e-9, 0e-9", end = "500e-9, 10e-9, 0", step =  "4e-9", dp_index= "0",
bufferCommand=True)
#ns.dp_save("filepath/DW_pos_%iter%.txt", dp_indexes=dp_index, bufferCommand=True)
```

```
        ns.Run()

    for r in range(N):
        for v in range(len(bulkDMIvalues)):
            bulkDMIvalue = bulkDMIvalues[v]
            for geometry in geos:
                for dmi in range(len(DMIS)):
                    RunSimH(geometry.strip(),DMIS[dmi],bulkDMIvalue*1e-6)
                    #Savefilename = 'filepath/DMI='+str(bulkDMIvalue)+'uJperm2'
                    #times,dw = AnalyzeData('rawdata',Savefilename+'.txt',totalT)

                    #plt.scatter(times,dw)
                    #plt.show()

def PlotMovement(times,dw,d,geometry,t1,t2,t3,t4,t5):
    totalT = t1+t2+t3+t4+t5
    plt.xlabel('time [ps]')
    plt.hlines(333,0,100,colors='gray',linestyles='dashed',label='equilibrium \n position')
    plt.axhspan(0,20,0,t2/totalT,color='orange',label='injector')
    plt.axhspan(0,20,(t2+t3)/totalT,(totalT-t5)/totalT,color='orange')
    plt.xlim(0,totalT)
    plt.plot([2],[60],marker=r'$\circlearrowleft$',ms=10,color='black')
    plt.plot([5+2],[60],marker=r'$\circlearrowleft$',ms=10,color='black')
    plt.plot([10+2],[60],marker=r'$\circlearrowleft$',ms=10,color='black')
    plt.plot([15+2],[60],marker=r'$\circlearrowleft$',ms=10,color='black')

    plt.plot([t2+t3+2],[60],marker=r'$\circlearrowright$',ms=10,color='black')
    plt.plot([t2+t3+5+2],[60],marker=r'$\circlearrowright$',ms=10,color='black')
    plt.plot([t2+t3+10+2],[60],marker=r'$\circlearrowright$',ms=10,color='black')
    plt.plot([t2+t3+15+2],[60],marker=r'$\circlearrowright$',ms=10,color='black')
    plt.subplots_adjust(left=0.15,right=0.95,bottom=0.15,top=0.85)
    plt.ylim(0,550)
    plt.ylabel('DW position [nm]')
    plt.scatter(times[:],dw[:]/1e-9,label='DMI: '+d+' , geometry: '+geometry)
    plt.legend(loc='upper left')

def PlotAll(geos,DMIS,t0,t1,t2,t3,t4,t5,N):
    totalT = t0+t1+t2+t3+t4+t5
    T2 = t1+t2+t3+t4+t5
    m = ['.','s','x','v']
    linestyles = ['-','--','-','--']
    c = ['black','gray','blue','turquoise']
    for r in range(N):
        for geometry in geos:
            for dmi in range(len(DMIS)):
                Savefilename = 'filepath/DWpositionOverTime-'+str(geometry)+'-'+str(DMIS[dmi]+'-run'+str(r))
                print(Savefilename)
                times = np.loadtxt(Savefilename+'.txt')[0]
                dw = np.loadtxt(Savefilename+'.txt')[1]
                plt.plot(times[:],dw[:]/1e-9,label=DMIS[dmi],color=c[dmi],linestyle = linestyles[dmi],linewidth=4)

    plt.xlabel('time [ps]')
    plt.hlines(333,0,100,colors='gray',linestyles='dashed')
    plt.axhspan(0,20,(t1)/T2,(t1+t2)/T2,color='orange')
    plt.axhspan(0,20,(t1+t2+t3)/T2,(T2-t5)/T2,color='orange')
    plt.xlim(t0,totalT)
    plt.plot([38],[9],marker=r'$\circlearrowleft$',ms=8,color='black')

    plt.plot([81],[9],marker=r'$\circlearrowright$',ms=8,color='black')
    plt.subplots_adjust(left=0.2,right=0.95,bottom=0.15,top=0.9)
    plt.ylim(0,500)
    plt.ylabel('DW position [nm]')

def PlotManyDMIS(bulkDMIvalues,geos,DMIS,N,Vs):
    #the times do vary depending on the material parameters, the anisotropy slope, and the excitation strength and length.
    # The following values are just examples!
    t0,t1,t2,t3,t4,t5 = 30,5,25,40,25,40 #for torque ex
    t0,t1,t2,t3,t4,t5 = 0,25,25,25,25,20 #for B field
    #Vs = [0.35,0.4]
    totalT = t0+t1+t2+t3+t4+t5
```

```
    T2 = t2+t3+t4+t5

    m = ['.','s','x','v']
    linestyles = ['-','--','-','--']
    col = pyla.cm.viridis(np.linspace(0,1,len(bulkDMIvalues)))
    c = ['black','gray','blue','turquoise']
    for r in range(N):
        for v in range(len(bulkDMIvalues)):
            bulkDMIvalue = bulkDMIvalues[v]
            for geometry in geos:
                for dmi in range(len(DMIS)):
                    Voltagevalue = Vs[dmi]
                    Savefilename = 'filepath/Data/'+str(geometry)+'-'+str(DMIS[dmi])+'-run'+str(r)
                    +'-DMI='+str(bulkDMIvalue)+'uJperm2-V='+str(Voltagevalue)

                    print(Savefilename)
                    times = np.loadtxt(Savefilename+'.txt')[0]
                    dw = np.loadtxt(Savefilename+'.txt')[1]
                    dw[dw<100e-9]=-100e-9

                    plt.plot(times-25,dw/1e-9,c=col[v])

    plt.xlabel('time [ps]')
    plt.hlines(333,0,100,colors='gray',linestyles='dashed')#,label='equilibrium \n position')
    plt.axhspan(0,20,0,25/T2,color='orange')#,label='injector')
    plt.axhspan(0,20,50/T2,75/T2,color='orange')
    plt.xlim(0,totalT-25)
    plt.plot([3],[9],marker=r'$\circlearrowleft$',ms=10,color='black')
    plt.plot([13],[9],marker=r'$\circlearrowleft$',ms=10,color='black')
    plt.plot([23],[9],marker=r'$\circlearrowleft$',ms=10,color='black')
    plt.plot([53],[9],marker=r'$\circlearrowright$',ms=10,color='black')
    plt.plot([63],[9],marker=r'$\circlearrowright$',ms=8,color='black')
    plt.plot([73],[9],marker=r'$\circlearrowright$',ms=8,color='black')
    plt.subplots_adjust(left=0.2,right=0.95,bottom=0.15,top=0.9)
    plt.ylim(0,500)
    plt.ylabel('DW position [nm]')


def PlotSpikes(times,dw):
    t0,t1,t2,t3,t4,t5,t6 = 20, 10, 20, 4, 2, 4 , 25
    Ttotal= t0+t1+t2#+t3+t4+t5+t6
    T2 = t1+t2#+t3+t4+t5+t6
    times = np.linspace(-25,35,len(dw))

    plt.plot(times+5,dw/1e-9,linewidth=5,color='black')
    plt.hlines(333,-25,30,colors='gray',linestyles='dashed',label='equilibrium',linewidth=3)
    #plt.hlines(224,0,100,colors='blue',linestyles='dotted',label='detector',linewidth=3)
    plt.axhspan(250,270,0,1,color='blue',alpha=0.2)#,label='injector') #the normal one is at 215
    plt.text(18,250,'detector',c='blue')

    plt.ylabel(r'$\mathcal{X}_\mathrm{DW}$ [nm]')
    plt.xlabel('t [ps]')

    plt.axhspan(0,20,0,8/25,color='orange')#,label='injector')
    plt.plot([4],[9],marker=r'$\circlearrowleft$',ms=10,color='black')

    plt.subplots_adjust(left=0.2,right=0.95,bottom=0.15,top=0.95)
    plt.ylim(0,400)
    plt.xlim(0,25)#Ttotal-25)


def PlotCompareTorqueExDMIs():
    file1 = './Data/IP-both-run0-DMI=200.0uJperm2-V=0.29.txt'
    times, dw = np.loadtxt(file1)[0],np.loadtxt(file1)[1]
    plt.scatter(times,dw/1e-9,color='blue')

    file2 = './Data/IP-both-run0-DMI=-200.0uJperm2-V=0.29.txt'
    times2, dw2 = np.loadtxt(file2)[0],np.loadtxt(file2)[1]
    plt.scatter(times2,dw2/1e-9,color='green')
```

```
    file3 = './Data/IP-None-run0-DMI=0uJperm2-V=0.43.txt'
    times3, dw3 = np.loadtxt(file3)[0],np.loadtxt(file3)[1]
    plt.scatter(times3,dw3/1e-9,color='black')
    plt.show()

def CompareStochasticAverage(bulkDMIvalues,geos,DMIS,N,Vs):
    t0,t1,t2,t3,t4,t5 = 35,5,25,40,25,40
    #Vs = [0.35,0.4]
    totalT = t0+t1+t2+t3+t4+t5
    T2 = t2+t3+t4+t5
    example = 'filepath/IP-both-run0-DMI=200.0uJperm2-V=0.295'
    times = np.loadtxt(example+'.txt')[0]

    DWs = np.zeros((N,len(times),2))
    DWnodmi = np.zeros((N,len(times)))

    for r in range(N):
        dw = 0
        Savefilename = 'filepath/str(r)+'-DMI=0uJperm2-V=0.41'
        dw = np.loadtxt(Savefilename+'.txt')[1]
        dw[dw<2e-7]=np.NaN
        #plt.scatter(times,dw)
        #plt.show()
        DWnodmi[r,:] = dw

    for r in range(N):
        for v in range(len(bulkDMIvalues)):
            bulkDMIvalue = bulkDMIvalues[v]
            for geometry in geos:
                for dmi in range(len(DMIS)):
                    dw = 0
                    Voltagevalue = Vs[dmi]
                    Savefilename = 'filepath/str(geometry)+'-'+str(DMIS[dmi])+'-run'+str(r)
                    +'-DMI='+str(bulkDMIvalue)+'uJperm2-V='+str(Voltagevalue)
                    print(Savefilename)
                    dw = np.loadtxt(Savefilename+'.txt')[1]
                    dw[dw<2e-7]=np.NaN
                    #plt.scatter(times,dw)
                    #plt.show()
                    DWs[r,:,v] = dw


    dwav = np.nanmean(DWs,axis=0)
    dwstd = np.nanstd(DWs,axis=0)
    dnod = np.nanmean(DWnodmi,axis=0)
    stnod = np.nanstd(DWnodmi,axis=0)



    plt.errorbar(times-t0+t1,dnod/1e-9,yerr=stnod/1e-9,xerr=None,elinewidth=0.2,linewidth=0.5,color='lightgray')
    plt.plot(times-t0+t1,dnod/1e-9,color='black',linewidth=3,zorder=3,label=r'$D=0$')
    plt.errorbar(times-t0+t1,dwav[:,0]/1e-9,yerr=dwstd[:,0]/1e-9,xerr=None,elinewidth=0.2,linewidth=0.5,color='lightblue')
    plt.plot(times-t0+t1,dwav[:,0]/1e-9,color='blue',linewidth=3,zorder=4,label=r'$D=200 \mathrm{\mu Jm}^{-2}$')
    plt.errorbar(times-t0+t1,dwav[:,1]/1e-9,yerr=dwstd[:,1]/1e-9,xerr=None,elinewidth=0.2,linewidth=0.5,color='lightgreen')
    plt.plot(times-t0+t1,dwav[:,1]/1e-9,color='darkgreen',linewidth=3,zorder=5,label=r'$D=-200\mathrm{\mu Jm}^{-2}$')

    plt.hlines(333,0,T2,colors='gray',linestyles='dashed')#,label=r'£x_0£')#)'equilibrium \n position')
    plt.axhspan(50,70,0,t2/T2,color='orange')#,label='injector')
    plt.axhspan(50,70,(t2+t3)/T2,(T2-t5)/T2,color='orange')

    plt.arrow(5,60,7,0,color='black',width=2,head_width=10,head_length=10)
    plt.arrow(90,60,-7,0,color='black',width=2,head_width=10,head_length=10)

    plt.xlim(0,totalT-t0+t1)
    plt.subplots_adjust(left=0.2,right=0.95,bottom=0.15,top=0.895)

    plt.rc('legend',fontsize=15)
    plt.legend(handlelength=1,handletextpad=0.5,loc=1,bbox_to_anchor=(1.0,0.53))
    plt.ylim(0,450)
    plt.ylabel(r'$\mathcal{X}_\mathrm{DW}$ [nm]')
```

```
    plt.xlabel('t [ps]')

def PlotDMIscanWithInset_IP(dmivalues):
    t0,t1,t2,t3,t4,t5 =  0,25,25,25,25,20
    totalT = t0+t1+t2+t3+t4+t5
    T2 = t2+t3+t4+t5
    #fig,ax1 = plt.subplots()
    fig2,ax2 = plt.subplots()
    col = pyla.cm.viridis(np.linspace(0,1,len(dmivalues)))
    DWmax1 = np.zeros(len(dmivalues))
    DWmax2 = np.zeros(len(dmivalues))
    for d in range(len(dmivalues)):
        Savefilename = 'filepath/DMI='+str(dmivalues[d])+'uJperm2'
        times = np.loadtxt(Savefilename+'.txt')[0]
        dw = np.loadtxt(Savefilename+'.txt')[1]
        r1min = np.amin(dw[int(len(dw)/4):int(len(dw)*0.6)])
        r2min = np.amin(dw[int(len(dw)*0.6):])
        r1max = np.amax(dw[int(len(dw)/4):int(len(dw)*0.6)])
        r2max = np.amax(dw[int(len(dw)*0.6):])
        eqpos = 333e-9
        if (abs(r1min-eqpos)>abs(r1max-eqpos)):
            DWmax1[d] = r1min
        else: DWmax1[d] = r1max

        if (abs(r2min-eqpos)>abs(r2max-eqpos)):
            DWmax2[d] = r2min
        else: DWmax2[d] = r2max

        ax2.plot(times-25,dw/1e-9,c=col[d])


    ax2.axhspan(0,20,0,25/T2,color='orange')#,label='injector')
    ax2.axhspan(0,20,50/T2,75/T2,color='orange')
    ax2.set_xlim(0,totalT-25)
    ax2.plot([13],[9],marker=r'$\circlearrowleft$',ms=12,color='black')
    ax2.plot([63],[9],marker=r'$\circlearrowright$',ms=12,color='black')
    ax2.set_ylim(0,430)
    ax2.set_ylabel(r'$\mathcal{X}_\mathrm{DW}$ [nm]')
    ax2.set_xlabel('t [ps]')

    plt.subplots_adjust(left=0.17,right=0.99,bottom=0.15,top=0.95)
    axins1 = ax2.inset_axes([0.53,0.22,0.47,0.22])
    axins2 = ax2.inset_axes([0.53,0.46,0.47,0.22])
    axins1.tick_params(axis='y')
    axins2.set_xticks([])
    ax2.text(35,150,r'$\mathcal{X}_\mathrm{DW} ^\mathrm{max}$ [nm]',rotation='vertical',size=15)
    ax2.text(63,35,r'$D$ [$\mathrm{\mu Jm}^{-2}$]',size=15)
    axins1.hlines(333,0,dmivalues[-1],colors='gray',linestyles='dashed')
    axins2.hlines(333,0,dmivalues[-1],colors='gray',linestyles='dashed')
    axins1.axhspan(380,460,0,10/T2,color='orange')
    axins2.axhspan(400,440,0,10/T2,color='orange')
    axins1.plot([0],[420],marker=r'$\circlearrowleft$',ms=12,color='black')
    axins2.plot([0],[420],marker=r'$\circlearrowright$',ms=12,color='black')
    axins1.tick_params(labelsize=15)
    axins2.tick_params(labelsize=15)

    for d in range(len(dmivalues)):
        axins1.scatter(dmivalues[d],DWmax1[d]/1e-9,color=col[d],marker='x')
        axins2.scatter(dmivalues[d],DWmax2[d]/1e-9,color=col[d],marker='o')


def PlotDMIscanWithInset_OOP(dmivalues):
    t0,t1,t2,t3,t4,t5 =  0,25,25,25,25,20
    totalT = t0+t1+t2+t3+t4+t5
    T2 = t2+t3+t4+t5
    #fig,ax1 = plt.subplots()
    fig2,ax2 = plt.subplots()
    col = pyla.cm.viridis(np.linspace(0,1,len(dmivalues)))
    #testdata = 'DWpositionOverTime-H=25.0MAperm-IP-both-run0-DMI=220.0uJperm2.txt'
    DWmax1 = np.zeros(len(dmivalues))#np.zeros(len(dmivalues),len(testdata[0]))
```

```
    DWmax2 = np.zeros(len(dmivalues))
    DWmax1t = np.zeros(len(dmivalues))#np.zeros(len(dmivalues),len(testdata[0]))
    DWmax2t = np.zeros(len(dmivalues))
    for d in range(len(dmivalues)):
        Savefilename = 'filepath/DMI='+str(dmivalues[d])+'uJperm2'
        times = np.loadtxt(Savefilename+'.txt')[0]
        dw = np.loadtxt(Savefilename+'.txt')[1]
        r1min = np.amin(dw[int(len(dw)/4):int(len(dw)*0.6)])
        r2min = np.amin(dw[int(len(dw)*0.6):])
        r1max = np.amax(dw[int(len(dw)/4):int(len(dw)*0.6)])
        r2max = np.amax(dw[int(len(dw)*0.6):])

        r1mint = np.argmin(dw[int(len(dw)/4):int(len(dw)*0.6)])
        r2mint = np.argmin(dw[int(len(dw)*0.6):])
        r1maxt = np.argmax(dw[int(len(dw)/4):int(len(dw)*0.6)])
        r2maxt = np.argmax(dw[int(len(dw)*0.6):])
        eqpos = 333e-9
        if (abs(r1min-eqpos)>abs(r1max-eqpos)):
            DWmax1[d] = r1min
            DWmax1t[d] = times[r1mint]
        else:
            DWmax1[d] = r1max
            DWmax1t[d] = times[r1maxt]

        if (abs(r2min-eqpos)>abs(r2max-eqpos)):
            DWmax2[d] = r2min
            DWmax2t[d] = times[r2mint]
        else:
            DWmax2[d] = r2max
            DWmax2t[d] = times[r2maxt]

        ax2.plot(times-25,dw/1e-9,c=col[d])


    ax2.axhspan(0,20,0,25/T2,color='orange')#,label='injector')
    ax2.axhspan(0,20,50/T2,75/T2,color='orange')
    ax2.set_xlim(0,totalT-25)
    ax2.plot([13],[9],marker=r'$\circlearrowleft$',ms=12,color='black')
    ax2.plot([63],[9],marker=r'$\circlearrowright$',ms=12,color='black')
    ax2.set_ylim(0,430)
    ax2.set_ylabel(r'$\mathcal{X}_\mathrm{DW}$ [nm]')
    ax2.set_xlabel('t [ps]')


    plt.subplots_adjust(left=0.17,right=0.99,bottom=0.15,top=0.95)
    axins1 = ax2.inset_axes([0.53,0.22,0.47,0.22])
    axins2 = ax2.inset_axes([0.53,0.46,0.47,0.22])
    axins1.patch.set_alpha(0.92)
    axins2.patch.set_alpha(0.92)
    axins1.tick_params(axis='y')
    axins2.set_xticks([])
    ax2.text(35,150,r'$\mathcal{X}_\mathrm{DW}^\mathrm{max}$ [nm]',rotation='vertical',size=15)
    ax2.text(63,35,r'D [$\mathrm{\mu Jm}^{-2}$]',size=15)
    axins1.hlines(333,0,dmivalues[-1],colors='gray',linestyles='dashed')
    axins2.hlines(333,0,dmivalues[-1],colors='gray',linestyles='dashed')
    axins1.axhspan(400,440,0,10/T2,color='orange')
    axins2.axhspan(400,440,0,10/T2,color='orange')
    axins1.plot([0],[420],marker=r'$\circlearrowleft$',ms=12,color='black')
    axins2.plot([0],[420],marker=r'$\circlearrowright$',ms=12,color='black')
    axins1.tick_params(labelsize=15)
    axins2.tick_params(labelsize=15)

    for d in range(len(dmivalues)):
        axins1.scatter(dmivalues[d],DWmax1[d]/1e-9,color=col[d],marker='x')
        axins2.scatter(dmivalues[d],DWmax2[d]/1e-9,color=col[d],marker='o')


def main():
    geos = ['IP'] # or OOP
    DMIS = ['both'] # for comparing the impact of DMI: ['both', 'onlyBulk', 'onlyHom', 'None']
```

```
    bulkDMIvalues = [200.0,-200.0]
    # np.arange(0,250,5,dtype=float) for scanning DMI range #250 max for OOP geometry, then collinear order is lost
    N = 1 #number ensemble members

    # call type of simulation and plotting functions here

if __name__ == '__main__':
        main()
```

REFERENCES

[1] S. Lepadatu, Boris computational spintronics—High performance multi-mesh magnetic and spin transport modeling software, J. Appl. Phys. **128**, 243902 (2020).
